# The Relationship between Reactive Oxygen Species and the cGAS/STING Signaling Pathway in the Inflammaging Process

**DOI:** 10.3390/ijms232315182

**Published:** 2022-12-02

**Authors:** Bárbara Andrade, Carlos Jara-Gutiérrez, Marilyn Paz-Araos, Mary Carmen Vázquez, Pablo Díaz, Paola Murgas

**Affiliations:** 1 Centro de Investigaciones Biomédicas (CIB), Facultad de Medicina, Escuela de Kinesiología, Universidad de Valparaíso, Valparaíso 2362905, Chile; 2 Instituto de Bioquímica, Facultad de Ciencias, Universidad Austral de Chile, Valdivia 5090000, Chile; 3 Center for Interdisciplinary Studies on the Nervous System (CISNe), Universidad Austral de Chile, Valdivia 5090000, Chile

**Keywords:** inflammaging, ROS, cGAS/STING pathway, cGAS, STING, immunosenescence, aging, DNA damage, macrophage, senescence, SASPs

## Abstract

During Inflammaging, a dysregulation of the immune cell functions is generated, and these cells acquire a senescent phenotype with an increase in pro-inflammatory cytokines and ROS. This increase in pro-inflammatory molecules contributes to the chronic inflammation and oxidative damage of biomolecules, classically observed in the Inflammaging process. One of the most critical oxidative damages is generated to the host DNA. Damaged DNA is located out of the natural compartments, such as the nucleus and mitochondria, and is present in the cell’s cytoplasm. This DNA localization activates some DNA sensors, such as the cGAS/STING signaling pathway, that induce transcriptional factors involved in increasing inflammatory molecules. Some of the targets of this signaling pathway are the SASPs. SASPs are secreted pro-inflammatory molecules characteristic of the senescent cells and inducers of ROS production. It has been suggested that oxidative damage to nuclear and mitochondrial DNA generates activation of the cGAS/STING pathway, increasing ROS levels induced by SASPs. These additional ROS increase oxidative DNA damage, causing a loop during the Inflammaging. However, the relationship between the cGAS/STING pathway and the increase in ROS during Inflammaging has not been clarified. This review attempt to describe the potential connection between the cGAS/STING pathway and ROS during the Inflammaging process, based on the current literature, as a contribution to the knowledge of the molecular mechanisms that occur and contribute to the development of the considered adaptative Inflammaging process during aging.

## 1. Introduction

Life quality improvement and progress in the health field have allowed humans to get old up to very high ages, even over 100 years old [1]. Consequently, people over 60 years old have significantly increased worldwide. Up to date, they are over 962 million, representing the world population, and this number is expected to double by the year 2050 (UN, 2017). In other words, 22% of the planet’s population will be over 60 years old by 2050, and 400 million people will be over 80 years old [1].

However, along with the rise in life expectancy of older people, it will also increase aging-related diseases, negatively impacting their health and quality of life [2]. Therefore, studying the different molecular mechanisms of natural aging and the diseases associated with aging is essential [1].

The age-related changes involve every cell all over the body, especially those from the homeostatic regulator systems such as the Immune System (IS) [3]. Along with aging, the cells from the IS deregulated their functions, for example, reducing the recognition and elimination of toxic molecules and pathogens, increasing in number, and becoming senescent or immunosenescent. The increase in pro-inflammatory secreted molecules and the alterations mentioned above is called Inflammaging. This Inflammaging process is considered a low-grade chronic sterile inflammation generated during aging [4]. It is also related to the rise in oxidative stress, driving progressive cellular component damage, such as proteins, lipids, and DNA [5].

The natural role of the immune cells is to produce several types of molecules to eliminate foreign and toxic dangerous agents as oxidant molecules and induce inflammation. However, over the years, the natural chronic inflammation triggers oxidative lesions, causing a progressive loss of normal cellular function and reducing the redox and inflammatory homeostatic capacity [5]. Consequently, during aging, there is an increase in the Reactive Oxygen Species (ROS). Alongside, there is also an increase in mitochondrial dysfunction due to the gradual increase in macrophage’s oxidative activity and other cell types of the innate immune system [6,7,8]. These events trigger a vicious cycle contributing to oxidative damage to mitochondrial and nuclear DNA. This phenomenon leads to the progressive deregulation of the immune system cell functions during aging, called Immunosenescence, and is considered part of the Inflammaging process [3,7,9,10].

The innate immune system cells have receptors or sensors that recognize intracellular nucleic acids as a signal of danger with infectious potential [11]. After the recognition process, these receptors produce intracellular signals to assemble inflammatory responses and protect the organism from infections [12,13]. Among the nucleic acid sensors, the universal DNA receptor, the cGAS protein, recognizes double strain DNA (dsDNA) in the cytoplasm and activates a signaling pathway involving the STING protein. The last one induces downstream transcriptional factors involved in gene expression that allow the release of cytokines, protein factors, and other inflammation-associated proteins and molecules. This enhances a protective inflammatory immune response [14,15,16,17]. The cGAS/STING signaling pathway also activates the senescence process when it recognizes damaged nuclear and/or mitochondrial DNA present in the cytosol, out of the normal compartment, the nucleus or mitochondria of the host cell, generating antitumor protection [18,19,20].

It is well known that senescence activation and increased oxidative stress are characteristic processes of the immune system aging or Inflammaging [21]. However, there is only speculative information about the cGAS/STING signaling pathway’s role in aging. There is extensive knowledge of its involvement in diseases associated with aging. Additionally, the cGAS/STING signaling pathway’s relation with the increase in ROS during aging is not fully understood. Thus, in this review, we aim to elucidate the potential relationship between the cGAS/STING signaling pathway and the accumulation of ROS during Inflammaging based on the recent literature. This understanding will provide a path for future investigations to contribute to the knowledge of new therapeutic strategies to understand the aging process and associated pathologies, such as premature aging, inflammatory, neurodegenerative, metabolic, and autoimmune diseases, and cancer.

## 2. Aging

The mechanisms responsible for healthy aging are closely related to the effective maintenance of the organism’s physiological, biochemical, and immunological functions [22]. However, the specific mechanisms associated with healthy aging and/or the loss of those, along with the adaptation to the environment through the life span, are still a matter of extensive study [23]. The following sections will describe some specific cellular mechanisms involved in aging.

### 2.1. Aging-Associated Cellular Mechanisms

Consequently, in geroscience, the discipline that seeks to understand the biology of aging and diseases, there is a complex relationship between factors that trigger aging and disease [15,24]. One of these fundamental molecular mechanisms, present in several types of diseases and aging, is chronic inflammation that induces progressive damage in the cellular macromolecules by generating stressor agents that affect practically all cells [15,24]. Among the molecules altered by chronic inflammation diseases and aging is genomic instability of the nuclear as well as the mitochondrial DNA, epigenetic alterations, together with the shortening of the telomeres, mitochondrial dysfunction, the progressive increase in cellular senescence, loss of proteostasis, nutrients dysregulation, alterations in intercellular communication and decrease in the stem cells. The above are considered hallmarks of the aging process [2,8].

#### 2.1.1. Genomic Instability

Genomic instability is the accumulation of genomic damage along the lifespan caused by exogenous (chemical, physical and biological) as well as endogenous (DNA replication mistakes, the action of ROS) agents [25]. However, cells have mechanisms to diminish the nuclear and mitochondrial DNA lesions. DNA reparation systems and cell cycle control mechanisms maintain cellular integrity [25,26,27]. During aging, these mechanisms are reduced and altered. For example, the expression of DNA repair enzymes is decreased, leading to DNA damage accumulation [28,29,30,31,32].

#### 2.1.2. Telomeres Shortening

Telomeres are structures localized at the end of the chromosomes made up of repeated DNA sequences [33,34]. They protect chromosomes from wrong recombination, degradation and recognition as damaged DNA [33,34]. Based on the telomeres length conservation, the telomerase enzyme performs this protective mechanism by reverse transcription of an RNA template [35]. During aging, several stressors, such as the damage induced by ROS, can be genotoxic, causing an accelerated shortening of telomeres [34]. Telomerase cannot compensate for this shortening, triggering cell functional alterations that lead to cell senescence activation [8,34].

#### 2.1.3. Senescence

Senescence is a physiological process that arrests cell cycle progression, along with phenotypic changes of senescent cells, such as the increase in the morphology and size of the nucleus. It aims to prevent the proliferation of damaged cells, a phenomenon that increases during aging [36]. These senescent cells accumulate in most tissues during aging, leading to progressive loss of tissue and organ function [37]. The senescent cells produce an increasing level of the Senescence-Associated Secretory Phenotype (SASP), a secretory phenotype of molecules that induce the senescence activation to endocrine and paracrine forms in the cells [38]. More on this topic will be detailed below.

#### 2.1.4. Mitochondrial Dysfunction

The mitochondrion is the cellular organelle that provides energy for proper cell functioning through oxidative phosphorylation and ATP synthesis [39]. During aging, mitochondrial function declines, resulting in reduced energy availability due to the deterioration of mitochondrial DNA (mtDNA) with a loss of homeostasis of mitochondrial enzyme functions, coupled with dysregulation of ROS production [7]. During life, there is an increase in mtDNA mutations with a deficiency of the damaged mtDNA removal mechanisms. The mtDNA repair machinery has to migrate from the nucleus to the mitochondria, and during aging, the expression of these proteins is reduced [8]. The mtDNA alterations induce mitochondrial dynamics dysfunction and contribute to cell deregulation during aging, increasing ROS production that potentiates the mtDNA damage, generating a vicious circle of mtDNA damage and a mitochondrial malfunction [7,8].

## 3. Inflammation to Inflammaging

Inflammation is a normal and necessary biological process in response to an injury or infection [40,41]; it is a highly regulated adaptive process, necessary and beneficial to the host when the damage is transient because it restores tissue homeostasis [40]. This process is characterized by an acute phase, where among other processes, leukocytes are recruited to the infected area mediated by cytokines, chemokines, and acute phase proteins [42]. Macrophages and phagocytic cells arrive at the infected site removing the inflammatory stimulus and/or the rest of the damaged cells to start the repairing process [42]. In the injury site, macrophages orchestrate the acute inflammation response and the repair/regeneration process releasing growth factors, chemokines, and cytokines, changing their phenotype from M1 or pro-inflammatory to M2 or anti-inflammatory, both with the capability to alter the local cellular environment and modulate the inflammatory immune response [42].

However, if the inflammation becomes chronic, the inflammatory cells can no longer remove the damage, lasting for weeks, months, and even years [43]. Chronic inflammation may develop several pathologies, such as cardiovascular, neurodegenerative, metabolic, autoimmune diseases, and even cancer.

With aging, a characteristic type of chronic inflammation occurs; this process is characterized by low-grade inflammation, sterile and persistent over time, which can progressively induce degeneration, dysregulation, and tissue damage, called Inflammaging [13,15].

**Figure 1:**
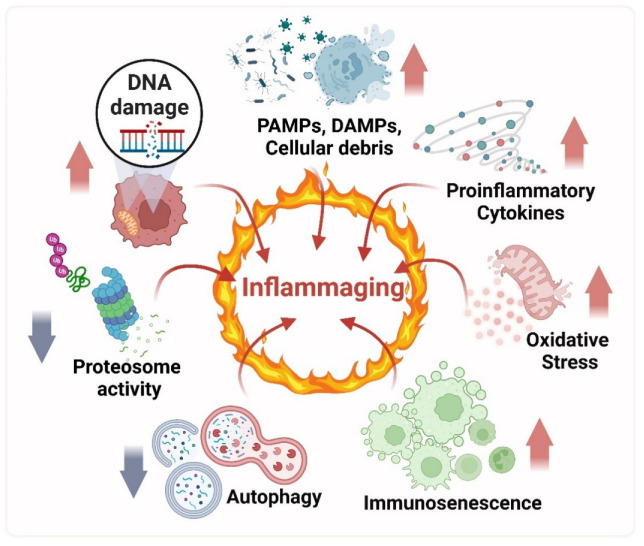
Mechanism of Inflammaging. The mechanisms that generate a progressive increase in the inflammatory state in the organism include the increase in the release of proinflammatory cytokines, the accumulation of oxidation products due to the increase in Reactive Oxygen Species (ROS), defects in the autophagy, and proteasome activity; both processes diminished. It is also generated by increased DNA damage in the nucleus and mitochondria, which produces cellular responses related to the induction of senescence in various tissues, including the immune system cells, called immunosenescence. The immunosenescence reduces the recognition and elimination of Pathogens-Associated Molecular Patterns (PAMPs), Damage-Associated Molecular Patterns (DAMPs), and cellular debris by phagocytic cells such as macrophages, and this induces an accumulation that also triggers Inflammaging.

The inflammaging concept describes a systemic low-grade chronic inflammation in the absence of infection, a process characteristic of aging and also associated with the chronic physiological stimulation of the immune system [4]. Inflammaging is triggered by a higher exposition to infectious and non-infectious antigens throughout the lifetime, becoming noxious in the elderly [4,13]. Several agents induce a persistent stress condition in the cellular microenvironment, which sustains a constant inflammation state in the organism [44]. Therefore, Inflammaging occurs due to an imbalance between pro- and anti-inflammatory mechanisms, leading to the loss of a proper inflammatory response of the immune system [13,41,45]. This imbalance can alter homeostasis regulation in other tissues of the organism [15,46]. A deregulated innate immune response turns into a chronic release of inflammatory cytokines, contributing to pathology development and the whole organism’s functional decline [13,47]. During the Inflammaging, a positive loop promotes chronic inflammation and increases the production of inflammatory mediators, which diminishes the innate and adaptive immune responses. This entails permanent cellular and molecular damage, accumulating over the years and triggering the activation of the immune system senescence or immunosenescence (Figure 1) [47,48]. One of the characteristics of immunosenescence is immune remodeling, characterized by a dysregulation in the ratio of T lymphocytes CD4^+^/CD8^+^ [49]. When this ratio is increased, it can be correlated with disease. However, in a person with healthy aging, this ratio is similar to younger adults [50]. In aging (nonpathological), there is an imbalance between Th1 and Th2 immune responses, the very elderly tend towards a Th2 profile, with both interferon (IFN)-g and IL-4 expressed at higher levels in CD4^+^ and CD8^+^ T cells of aged as compared to younger persons after in vitro stimulation [51,52]. However, there is also this delicate balance between inflammation and Inflammaging. This Immune adaptation is the process that occurs when leukocytes that have been exposed to environmental stimuli can modify their properties so that they can influence their future response to that stimulus or another. This response is dynamic and reversible depending on the evolution of the environmental conditions. Even though the changes may be reversible, these adaptative phenotypes may persist in varying frame times [48,53].

The cells and their macromolecules are physiologically under constant damage and repair processes; this phenomenon raises cellular debris products or “cellular garbage” [45]. However, over the years, residue removal mechanisms by macrophages progressively decrease, leading to an accumulation of these molecules [15,45]. Additionally, molecules from the gut microbiota product of the dysbiosis and loss of gut permeability, together with persistent or recurrent pathogen infections by, for example, Epstein Barr virus, Cytomegalovirus, HIV, Coronavirus, and others, can also trigger a continuous immune inflammatory response along time, which lead to tissue damage [12,15,45,54].

The main initiators of the Inflammaging are the host’s misplaced, altered, and damaged molecules that accumulate in different tissue aging [45]. The inflammation occurs in response to endogenous mediators released by stressed, damaged, or malfunctioning tissues [40,55]. Some immune receptors expressed on the plasma membrane and in the cytoplasm of the innate immune cells, such as the macrophages, activate signaling pathways that increase the inflammation mediators [13]. Chronic inflammation is triggered mainly by Damage-Associated Molecular Patterns (DAMPs), endogenous molecules such as proteins and DNA, which can constantly be altered, damaged, or misplaced inside the cell [13,45]. Among those endogenous molecules mentioned, it is suggested that damaged DNA and/or located outside its regular cellular compartments, such as the nucleus or mitochondria, can be a significant enhancer of the Inflammaging as well as the innate immune system chronic activation, inducing the cellular response to damage and an increase in the inflammatory cytokines releasing, for example from the activated macrophages [45,56].

Consequently, chronic inflammation implies the activation of molecular pathways with the altered production of several cytokines, effector molecules, and a tissue response that are common to many pathologies related to aging (Figure 1) [12,15]. Therefore, Inflammaging represents a higher vulnerability to infections and susceptibility to developing age-related diseases, such as arthritis, asthma, diabetes, atherosclerosis, autoimmune diseases, neurodegenerative diseases, and cancer [15,40,42]. This way, Inflammaging is highlighted as the leading risk factor for chronic morbidity, functional impairment, and mortality of aging [8,12,13,15,47].

### The Macrophages: Leading Role Cells of the Inflammaging Process

During Inflammaging, there is a decline in the effective elimination of pathogens by the innate immune system due to a reduced ability to recognize and eliminate pathogens [3]. Then, the IS compensates for this insufficient, increasing the number of macrophages and other phagocytic cells that lead to proinflammatory cytokines production in response to pathogenic microorganisms. These cytokines amplify the inflammatory response and induce a generalized inflammatory process in the whole organism [56]. This chronic activation of the innate IS has as a protagonist the macrophage [4,46,56]. The macrophages usually monitor the state of the cells in the tissues where they reside through the binding of the Pathogen-Associated Molecular Patterns (PAMPs) or DAMPs to the Pattern Recognition Receptors (PRRs), which trigger intracellular signals that induce the release of inflammatory cytokines in response to the tissue homeostasis alteration at the site of damage [11,44,57,58]. The macrophage can phagocytose the PAMPs and DAMPs to eliminate them. In addition, the increase in ROS production in the oxidative burst process, which includes several highly toxic reactions, can destroy the harmful agent [40,59].

ROS generation is one primary cellular mechanism that occurs during aging due to chronic exposure to stressor agents and excessive macrophage stimulation [60]. In this way, during Inflammaging, ROS production is exacerbated, as well as cytokines generation, all of which alter the phenotypes of the macrophages surrounding cells and induce the normal tissue function detriment [59]. Therefore, the macrophages promote the inflammatory process during aging, making it extensive and chronic [15].

The increase in ROS production during Inflammaging gives rise to damage to immune as well as non-immune cells, which accumulates in all the tissues with age [61,62].

## 4. Reactive Oxygen Species (ROS)

ROS are produced physiologically by activating various metabolic pathways and regulating essential cellular functions [63]. These molecules have a dual role in health and disease since they also contribute to the pathophysiology of various chronic inflammatory diseases, cardiovascular and neurodegenerative diseases, and cancer, among others [64].

ROS are substances necessary for proper cell function and play an essential role in cell signaling, inducing survival mechanisms, and maintaining homeostasis [65,66,67]. For example, they play an essential role in immune defense, vascular tone, and signal transduction [66]. However, excessive ROS production constitutes a risk for the cells. Therefore, the maintenance of cellular homeostasis of ROS production must be regulated [66]. This regulation is achieved under physiological conditions since there is a balance between the generation of ROS and their elimination by antioxidant defenses [3].

The primary antioxidant function is maintaining ROS concentrations between the beneficial ranges of the organism and maintaining homeostasis [68]. This process is called eustress (or “positive stress”), where normal cellular responses such as proliferation, differentiation, migration, and angiogenesis are promoted [68]. ROS levels slightly above eustress lead to stress and adaptive responses. Meanwhile, ROS concentrations above this level lead to distress, which has negative consequences for the cells, such as inflammation activation, oxidative damage, cell cycle arrest, tumor development, and cell death [68].

Oxidative damage is an alteration in the prooxidant-antioxidant balance in favor of prooxidant substances. This leads to an interruption of redox signaling and its control and/or molecular damage [68]. This event, known as oxidative stress, can occur from an increase in ROS and/or a decrease in homeostatic responses of antioxidant defense [63]. ROS can react with almost every macromolecule inside the cell, such as proteins, lipids, and nucleic acids, and the cumulative effect of these deleterious interactions eventually leads to cell death [63,65,66]. Among the biomolecules affected by ROS, the damage suffered by DNA is the most significant that the cell can suffer since it can affect both the integrity and the regulation of genes, so it must be repaired through mechanisms cells to prevent mutagenesis, maintain cell genomic stability, and protect normal biological functions [63,65,69].

Despite the protective mechanism cells possess, cell damage levels can be increased under oxidative stress due to increased ROS from various exogenous and endogenous sources (Table 1) [68,70].

**Figure 2:**
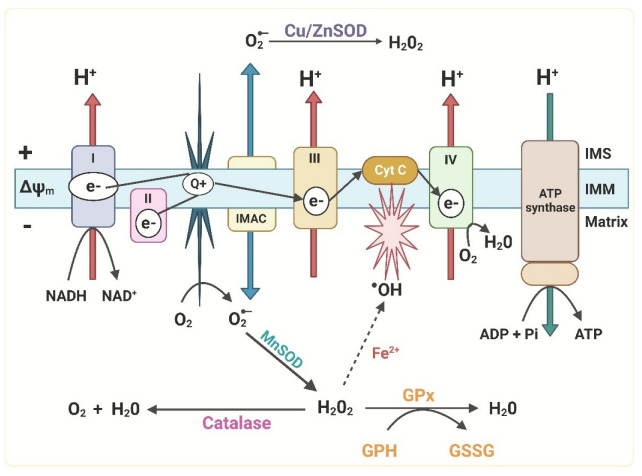
Generation of ROS in the Electron Transport Chain (ETC) and mitochondrial antioxidant defenses. During normal metabolism, small amounts of electrons leak from mitochondrial complexes I and III, generating O_2_•^−^ production. H_2_O_2_ can interact with metal ions through the Fenton reaction (dotted line) and cause a highly reactive compound, •OH, which can damage DNA, proteins, and lipids. IMS: intermembrane space; IMM: inner mitochondrial membrane; NADH: Reduced Nicotinamide Adenine Dinucleotide; NAD^+^: Oxidized nicotinamide adenine dinucleotide; MnSOD: Manganese superoxide dismutase; Cu/ZnSOD: Copper/zinc superoxide dismutase; ADP: Adenosine diphosphate; Pi: inorganic phosphate; ATP: Adenosine Triphosphate; Q^+^: coenzyme Q or ubiquinone; IMAC: Inner membrane ion channel; Cyt c: Cytochrome c; GPx Glutathione peroxidase; GPH: Glutathione; GSSG: Oxidized Glutathione; ΔΨm: membrane potential.

The primary endogenous source of ROS is mitochondria [65]. The principal function of the mitochondrion is oxidative phosphorylation, which is a vital process for providing energy to the cells in the form of ATP. Nevertheless, this mechanism is also one of the primary sources of ROS production and release inside the cell. Usually, this phenomenon is counteracted by antioxidant defenses, which are found at higher levels in mitochondria and play a fundamental role in maintaining cellular redox status [80] (Figure 2).

**Figure 3:**
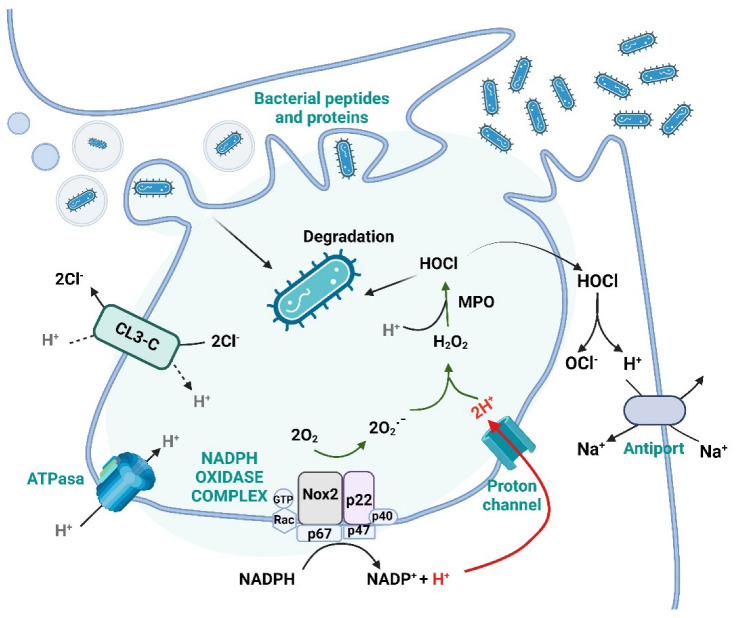
ROS formation during the oxidative burst in phagocytic cells. After the phagocytosis of microorganisms, the activation of the enzyme NADPH Oxidase (NOX) facilitates ROS production inside the phagosome which will kill the pathogen. MPO: myeloperoxidase enzyme; HOCl: hypochlorous acid; Cl^−^: chlorine anion; H_2_O_2_: hydrogen peroxide; O_2_•^−^: superoxide anion; O_2_: molecular oxygen; NADPH complex: Rac-p47-p67-gp91-p40-p22 subunits.

ROS are produced by the action of NADPH oxidase (NOX) enzymes [68]. It is suggested that mitochondria and NOXs, under conditions of oxidative stress, can stimulate each other so that mitochondrial ROS could activate NOXs and vice versa, generating a vicious cycle [80]. Mitochondria and NOX enzymes increase ROS in cells of the IS, which play essential roles in their response (Figure 3) [68,81].

### 4.1. ROS and Its Function in the Immune System

ROS are involved in various aspects of the IS, such as host defense, immune cell activation, interaction, and immunosuppression [81]. Regarding immune protection, innate cells such as neutrophils, macrophages, and Natural Killer are the first line of defense and release ROS to damage and eliminate pathogenic microorganisms and infected cells. In addition, reactive nitrogen species are added to ROS in the immune defense, which is generated once the NOS enzyme is activated, producing nitric oxide (NO) during the inflammation processes [82]. During defense against pathogens, the activation of NOX results in a rapid increase in oxygen consumption, which is known as an “oxidative burst” also called a “respiratory burst” (Figure 3) [65,81].

Within the ROS generated by the immune system, H_2_O_2_ readily diffuses through cell membranes and acts as a second messenger in different signaling pathways of immune cells, which allows them to be regulators of this response [83,84]. Although ROS are essential for the immune response and other cellular functions, when the redox balance is altered, oxidative damage is generated, which can lead to cell dysfunction and death, both in immune and non-immune cells, which contributes to the appearance of aging [80].

### 4.2. ROS Role in Aging

ROS are generated in greater quantity during aging due to continuous exposure to stressful agents and the constant stimulation by proinflammatory cytokines of NOX [60]. There is also a decrease in the effectiveness of enzymatic antioxidant defenses, resulting in the loss of homeostatic redox balance and the generation of oxidative damage in the organism’s cells [85,86,87].

“The free radical theory of aging” postulated by Harman (1956) [10], relates the generation of ROS with the gradual accumulation of macromolecular damage and the consequent loss of cellular function as the primary mechanism underlying aging [7,69,86]. These molecular damages accumulate and affect different cell types and tissues [3,8,88]. After this initial theory, the “mitochondrial oxidative stress” theory proposed by Miquel (1980) [89] evolved, which suggests that during aging, the increase in oxidant products, as well as the progressive failure of antioxidant mechanisms, cause damage to macromolecules, which accumulates with age and is closely related to the decline in cell function [3,90]. This last theory proposes the existence of a redox control/uncontrol in aging, where changes are produced in the activation/deactivation of redox-sensitive proteins, with the contribution of ROS to the activation of cellular senescence, which translates into alterations in genes and mitochondrial membranes of differentiated post-mitotic cells, with progressive loss of immune function, and of other tissues and organs [3,90].

The increase in ROS released from the mitochondria is due to the greater genomic instability of mtDNA, which lacks its enzyme system for DNA repair, which makes them even more susceptible during aging [91,92,93,94]. Damage to mtDNA translates into the deregulation of mitochondrial function, with an increase in the production of ROS, which leads to a vicious cycle of damage and mutations in mtDNA that alter the role of the respiratory chain, generating more ROS during aging [89,95,96,97,98]. This constant increase in ROS in the mitochondria induces energy depletion and contributes to progressive cell damage and the activation of stress pathways that will generate senescence or cell death [7,89,91,99]. Similarly, the overproduction of ROS also induces nuclear DNA (nDNA) damage, which causes progressive deregulation in cell functions, accelerating senescence and, consequently, cell death [3,7,8,89,98,100,101].

The damage generated by increased levels of ROS in specialized cells, such as those that are part of the IS, has been seen to contribute to the development of Inflammaging [3,102].

## 5. Activation of Cellular Death Mechanisms against DNA Oxidation Damage

The oxidative damage suffered by biomolecules such as DNA in immune and other cells allows the activation of DNA damage repair mechanisms and intracellular signaling pathways or damaged DNA response (DDR) [103,104,105,106,107,108].

Unlike other biomolecules, damaged DNA cannot be replaced, so proper cell function depends on its repair [109]. DNA lesions are constantly generated due to ROS from cellular mitochondria and oxidative bursts in cells such as macrophages [110]. Both nDNA and mtDNA are affected by ROS increase to combat oxidative damage, and they have repair mechanisms analogous to those found in the nucleus [92,94]. However, mtDNA oxidative damage occurs more frequently than nuclear damage due to the source of ROS production, and the mitochondria contain naked DNA without histones [94].

During processes of genotoxic stress and excessive DNA damage (as in conditions of oxidative stress), DNA repair is altered, and DNA destabilization is accelerated, generating the formation of micronuclei [25,111]. During aging, the enzymes of the repair mechanisms gradually decrease in expression, and the absence of repair of oxidized DNA generates the accumulation of oxidative lesions, transforming DNA into unstable and mutagenic [29,86,92,112]. This triggers the additional generation of ROS from the mitochondria and other organelles, producing a positive loop of increased genome instability, oxidative damage, and mitochondrial dysfunction, which affects all cell functions and leads to aging [7,89,92,112,113,114,115].

**Figure 4:**
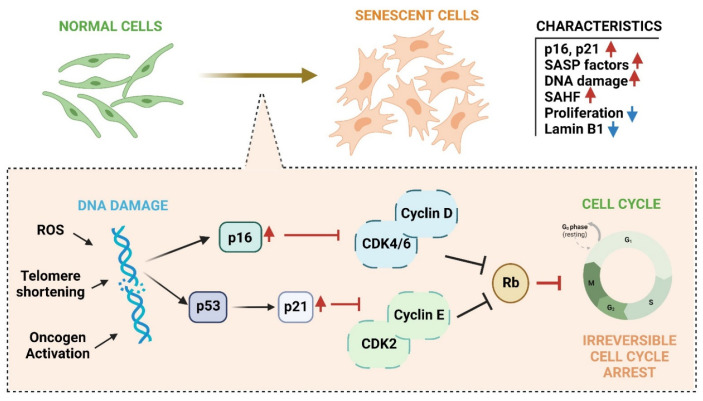
Cell cycle arrest pathways that are activated during senescence. Various stressors, such as irradiation, telomere shortening, oncogene activation, and increased ROS, generate DNA damage that induces DDR and activates the p53-p21 and p16 pathways, which inhibit specific cyclin-dependent proteins. Rb inhibitory kinase prevents cycle progression by inhibiting EF2 (not shown in the image), which leads to irreversible cell cycle arrest in senescent cells. In addition, senescent cells present other characteristics such as decreased nuclear protein Lamin B1, senescence-associated heterochromatin foci (SAHF) formation, increased DNA damage, and increased SASPs.

In addition to the DNA repair machinery, genome stability is safeguarded by DNA damage response (DDR) signaling pathways [25]. In this way, when DNA lesions are not repaired in time, either due to extensive or persistent damage or due to a deficiency in substrates for repair, and even because the repair mechanisms are overwhelmed, DDR is generated [36,105,116]. DDR can also be initiated by DNA damage produced by endogenous metabolites such as ROS and induce processes such as cellular senescence or apoptosis [25,104,109]. When DNA damage is persistent and/or severe, and the repair mechanisms fail to counteract the damage, the cell activates the senescence and Regulates Cell Death (RCD) [36,105,116]. Both cellular senescence and RCD are present throughout life, and their function is protective against the accumulation of damaged, potentially tumorous, or malignant cells [117]. It has been postulated that apoptosis, an RCD, is generated as a cellular response to extensive stress, while senescence is activated by minor damage [118]. The activation of senescence is triggered by the persistence of the DDR or stress signals, which leads to the activation of signaling pathways mediated by p53 and/or p16 proteins, and, therefore, to cell cycle arrest irreversibly (Figure 4) [36,117,119]. However, the factors that influence the “decision” made by the cell to activate one or the other process are still not completely understood [36,119]. However, it has been shown that the type of cell, as well as the intensity, type, and extension of the signal that triggers the damage, are determining factors in cell fate and also in the efficiency of repair [36,115,119,120,121,122].

During aging, the increase in damage by ROS makes these protective mechanisms fundamental and triggers a progressive cellular senescence activation [117].

## 6. Cellular Senescence and Senescence Associated Secretory Phenotypes (SASPs)

Hayflick & Moorhead (1961) [123] described cellular senescence for the first time. They observed that human cells cultured in vitro divided finitely. This limitation in proliferation was related to the shortening of telomeres in each cell cycle, calling it “replicative senescence” [37]. As mentioned, senescence occurs in response to stress and telomeric and non-telomeric DNA damage. This damage is caused by various genotoxic and non-genotoxic stressors, which generate disturbances in chromatin organization, such as activation by oxidative stress [36,37,124,125].

Depending on the biological context, senescence can be beneficial or detrimental to the organism. For example, during the embryonic stage, it is essential for the morphogenesis process and the proper development of the embryo [119,126]. During adulthood, senescence is a potent tumor suppressor mechanism and participates in wound repair and regeneration [36,117,119]. Most senescent cells present a set of changes in their behavior, morphology, structure, and cell function, known as the senescent phenotype [36]. In most senescent cells, these changes include morphological changes, growth arrest, resistance to cell death signals, secretion of a defined profile of soluble molecules, and alterations in gene expression (Figure 4) [36]. Mainly, senescent cells present dysfunctional mitochondria with higher levels of ROS [90]. Unlike quiescent cells reversibly arrested in phase zero G0 of the cell cycle, cells undergoing senescence lose the ability to divide but remain metabolically active [117,127].

In conjunction with extensive chromatin alterations and inflammatory mediators release, senescence slowly progresses from an early stage to completely irreversible and phenotypically complete senescence [125]. At the beginning of the propagation of senescence, the release of secreted proinflammatory cytokines, chemokines, growth factors, and other immune modulators are necessary, which are known as SASPs or senescence message secretome (SMS) [121,128,129,130,131]. The released SASPs mainly include the cytokines, Interleukin-6 (IL-6) and Interleukin-8 (IL-8), which act in an autocrine and paracrine manner to reinforce cell cycle arrest in distant and adjacent cells, generating an amplification of senescence in tissues [32,132,133]. SAPs are essential to communicate their commitment to other cells and thus stimulate tissue repair when damage has occurred, recruiting the immune system cells and inducing local inflammation [90]. In addition, SASPs are promoters and attract macrophages, Natural Killer cells, and T lymphocytes, to activate the elimination of senescent cells, a process called “senescence surveillance” [124,125,134]. However, during aging, the higher levels of SAPs and the reduction in the phagocytic capacity of macrophages generate a progressive accumulation of senescent cells in all tissue and organs [37,46,125] by insufficient cell replacement, as a consequence of a reduction in the population of stem cells, as well as a deficiency in the elimination of damaged cells [8,37,113,115,125,135]. The accumulation of senescent cells generates a progressive increase in circulating SASPs with a progressive loss of function in old organs and tissues, with adverse consequences for develop of diseases and aging-related morbidity [37]. This phenomenon promotes a proinflammatory environment that contributes to the generation of Inflammaging [12,13,15,46,99].

### 6.1. Senescence Molecular Pathways Mediated by ROS

Cellular senescence has multiple triggers and activation pathways, with mechanisms varying by cell type and other environmental circumstances. The main known mechanisms are those involved in “damage-induced senescence” [117]. Those that include subtypes such as “replicative or telomeric damage senescence”, “DNA damage-induced senescence”, “stress-induced senescence” and “oncogene-induced senescence” are all related to the increase in ROS [105,106,108,117,136]. In other words, the increase in ROS generates oxidative damage to DNA, which activates several senescence signaling pathways [37,90,105].

ROS are intrinsically related to activating the signaling pathways that induce inflammatory processes [99]. Specifically, ROS regulates the activation of transcriptional proteins associated with proinflammatory genes, such as the Nuclear Factor Kappa Light Chain Enhancer of Activated B Cells (NFκB) [137]. NFκB is the master transcriptional factor that regulates the transcription of numerous genes related to cell cycle control, inflammation, immune response, and oxidative stress [3,138,139]. NFκB and its inhibitors Inhibitory Kinase (IKK) and IkB proteins form a redox-sensitive complex; the increase in ROS induces IKK, which phosphorylates IκB, releasing the p65/p50 heterodimer, the components of NFκB. IkB is cleared by the ubiquitin-proteasome system, allowing NFκB to translocate into the nucleus and initiate transcription of several proinflammatory genes [68,104,140,141]. Among its target genes are cytokines such as IL-6, IL-1, TNF-α, the p53 and p16 proteins (regulators of the cellular cycle), growth factors, and molecules associated with cell adhesion [107,108,121,124,136,138,142]. Various molecules from cellular stress, DNA damage, and inflammation, specifically inflammatory cytokines, interact with receptors on the cell surface of immune cells. This interaction initiates intracellular signaling cascades that activate transcription factors that regulate/deregulate chronic inflammation in various tissues during aging, including NFκB [15,143].

**Figure 5:**
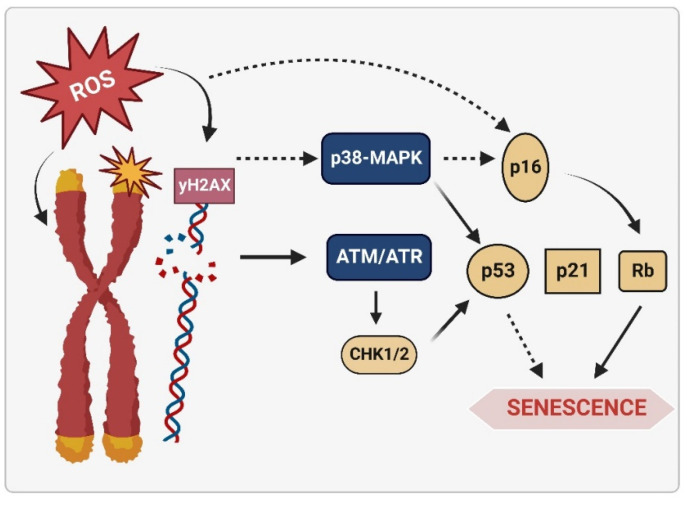
Signaling pathway in response to DNA damage and activation of ROS-mediated senescence. ROS generates telomere damage and DNA oxidation, which activates DDR through p53 signaling for cell cycle arrest. This is recognized by the ATM/ATR sensor proteins, which orchestrate a signaling network by phosphorylating messenger proteins that generate p53 activation. p53 subsequently induces transcription of p21, which activates downstream Rb, and Rb, in turn triggering senescence by stopping transcription of E2F genes and, ultimately, cell cycle arrest or senescence. ROS, in addition to starting DDR by oxidative DNA damage, generates the activation of p53 and p16 for cell cycle arrest through the p38MAPK protein pathway.

As mentioned, DNA damage activates senescence through the DDR signal, where stress-induced NFκB participates and allows the generation and release of SASPs [124,128]. NFκB is the main transcription factor that accumulates in the chromatin of senescent cells and participates in the positive regulation of SASPs, promoting the release of inflammatory mediators associated with senescence and, therefore, the process as such [124,128]. Various signaling pathways converge to activate NFκB in senescent cells, such as the cGAS/STING signaling pathway (described below) and the p38/MAPK, among others (Figure 5) [117,130,144]. The p16-pRb pathway is also activated through mitochondrial dysfunction mediated by the p38MAPK pathway and increased ROS production (Figure 5) [125]. In addition, activated p21 has been shown to initiate signaling through p38/MAPK and TGFβ, leading to mitochondrial dysfunction and a subsequent increase in intracellular ROS [130]. Higher levels of ROS generate nuclear DNA damage and, therefore, more significant long-term damage, irreversibly maintaining proliferation arrest during the establishment of the senescent phenotype [130].

During aging, there is an increase in damaged cells and the consequent activation of NFκB in the tissues, which is detected by IS cell recognition receptors, which downstream trigger the activation of NFκB and which in turn increases Inflammaging [3,12,45] propose the theory of “oxidation-inflammation” as the principal agent that affects the communication between the cells of the immune system with the rest of the organism and tissues, which alters the general homeostasis during aging. This theory is based on the intracellular increase in NFκB in immune cells given by ROS, which generates the most significant increase in inflammatory mediators and release of oxidant compounds, producing chronic oxidative stress [3].

### 6.2. The Senescence of the Immune System: Immunosenescence

During Inflammaging, innate and adaptive immune cells are induced to progressively lose their ability to regulate their redox and inflammatory balance [5]. The deregulated and inadequate immune response resulting from increased senescence or cell cycle arrest is called “Immunosenescence” [48,102,145]. The main features of Immunosenescence include a reduced ability to respond to new antigens, an increase and reduction in the same immune cells, altered memory responses, and activation of Inflammaging [48,146].

Immunosenescence is considered one of the causes of the generation of Inflammaging throughout life [15,48]. Immunosenescence and Inflammaging are the main changes the immune system experiences in aging [48,62]. These changes would induce pathologies such as an increase in infections, cancer, autoimmune, and chronic inflammatory diseases, all of which are present in a high percentage of the older adult population [48,147].

The changes experienced by the senescent immune system include modifications of innate IS. For example, cells such as Natural Killer increase in number, but their cytotoxic capacity decreases [148]. Dendritic cells decrease their phagocytic and antigenic presentation capacity and suffer alterations to migrate to the sites where they are required [48]. Senescent neutrophils show a decrease in superoxide production [47]. While in monocytes and macrophages, their ability to present antigens is reduced, given the reduction in class of major histocompatibility complex type II (MHC II) expression, and their phagocytic capacity is also reduced [102]. Furthermore, these cells increase in number in circulation to compensate for the deficiency in pathogen recognition. Their state of differentiation is predominantly towards the M1 phenotype, where an increased release of ROS and proinflammatory cytokines is observed. This makes them the main protagonists and contributors to the Inflammaging process during life and aging [47,149]. Senescent macrophages release SASPs, and the accumulation of ROS damage leads to decreased autophagy, with a reduction in the senescent cell elimination by phagocytosis, aggravating inflammation during this process [149].

The cells of the adaptive immune system, when they become senescent and aged, also alter their functions. For example, naive T lymphocytes decrease in number due to progressive thymic involution and atrophy [47,102]. An increase in the number of mature CD8^+^ and CD4^+^ T cells is generated, in both cases memory and effector [150]. In addition, in these T lymphocytes, there is an increase in the production and release of inflammatory cytokines and an alteration in intracellular signaling due to the decrease in the expression of T lymphocytes receptors [47,151]. In the case of the B-lymphocytes, their number is maintained during aging—however, their ability to secrete immunoglobulins and cytokines decreases. Therefore, the humoral and cellular memory to recognize new infectious agents is reduced in quantity and quality [48].

Among the mechanisms that induce immunosenescence are the recognition of stress stimuli, such as damaged DNA and ROS increase, and the sequent activation of signaling pathways that generate changes in the cellular immune response during life. Among the receptors that detect these stress molecules and trigger immunosenescence are the PRRs [44,152,153].

## 7. Patterns Recognition Receptors and Damaged DNA

Various types of Pattern Recognition Receptors (PRR) families are located both in the plasma membrane and membranes of cytoplasmic vesicles and endolysosomes of cells [11,44,57,153]. The most studied of them are Toll-Like Receptors (TLRs). However, there are many others such as the C-type lectin receptors (CLRs), retinoic acid-inducible gene (RIG-I) receptors (RLRs), nucleotide-binding oligomerization domain receptors (NOD) (NLRs), and cytosolic DNA receptors (CDRs) [11,44,57,153].

By recognizing molecular patterns in microorganisms, the PRRs expressed in IS cells activate intracellular signaling cascades to generate the immune response [11,44,57]. The signaling pathways activated by PRRs converge in the nuclear translocation of classic transcriptional immune response factors, such as NFκB and the Interferon Regulatory Factors (IRF). The IRF is a family of transcriptional factors with more than ten types; each one participates in various processes with biological effects such as the induction of a response against pathogens, the signaling of cytokines, cell cycle regulation, and hematopoietic development [154]. Both NF-kB and IRF factors induce the synthesis and secretion of inflammatory molecules such as cytokines, chemokines, and several types of Interferons (IFNs), all molecules that allow the recruitment of immune cells, the increase in inflammation at the site of damage or infection, and subsequently, the regulation of the resolution of aggression, to allow tissue repair [155].

**Figure 6:**
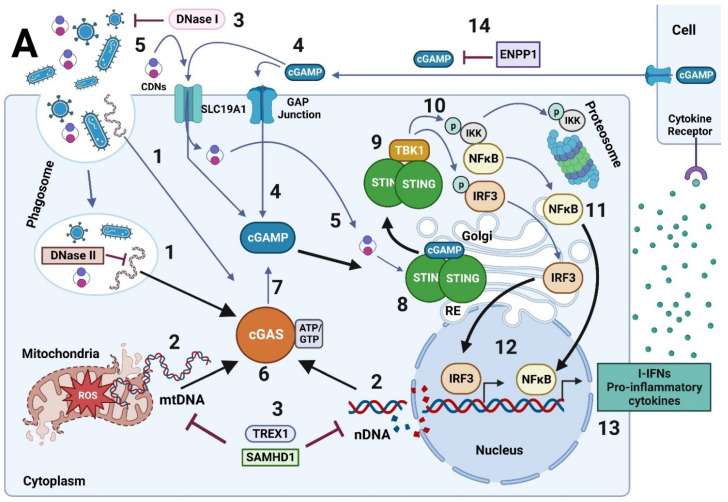

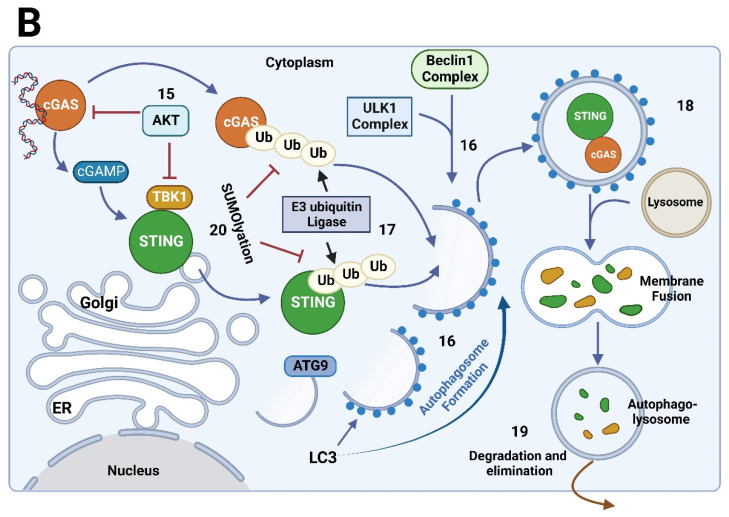
Mechanisms of activation and regulation of the cGAS-cGAMP-STING signaling pathway. (**A**) Source of cytoplasmic DNA: (1) DNA can come from external sources such as pathogenic DNA and virions (and be ingested for digestion in endolysosomes, which can release it into the cytosol) (2) DNA can come from intrinsic sources such as damaged self-DNA, which is released into the cytoplasm after damage to nuclear DNA and/or mitochondrial DNA. The latter moves to the cytoplasm after permeabilization of the mitochondrial outer membrane during mitochondrial stress, which can release oxidized DNA. (3) DNases such as TREX1, DNase II, and SAMHD1 degrade DNA and limit its recognition by DNA sensors. (4) cGAMP can be transferred between neighboring cells by various mechanisms, including extracellular vesicles and intercellular GAP junctions. (5) Furthermore, various CDNs (including cGAMP) can be exported by the reduced folate transporter or SLC19A1. (14) cGAMP can be hydrolyzed by ectonucleotide pyrophosphatase/phosphodiesterase family member 1 (ENPP1). (6) The cGAS/STING signaling pathway is activated with the recognition of cytoplasmic DNA given by cGAS, which produces (7) the second messenger, cGAMP. Subsequently, cGAMP binds to STING (8), which dimerizes and exits the ER towards the Golgi. STING undergoes oligomerization and palmitoylation and then binds to (9) TBK1 to form a complex. (10) TBK1 then phosphorylates IRF3 and IκB kinase (IKK) in parallel. (11) IKK phosphorylates the inhibitor of NFκB (IκB) to generate the release of NFκB. (12) Subsequently, both transcription factors, IRF3 and NFκB, translocate to the nucleus and generate the transcription of (13) IFN I and inflammatory cytokines, respectively. (**B**) (15) The inactivation of cGAS and TBK1 is mediated by AKT phosphorylation and the degradation of the action of several types of caspases. (16) The degradation of cGAS and STING is generated after the formation of (16) Beclin and ULK1 complex to activate the (16) autophagosome formation (17) previous ubiquitination of cGAS and STING mediated by E3-ubiquitin Ligase. (18) STING, after being activated, induces ER stress and ULK1, together with Beclin-1, initiates the formation (18) of the autophagosome with the participation of ATG9 and LC3 proteins, where cGAS and STING will be (19) degraded. (20) One of the mechanisms that inhibit the degradation of cGAS and STING proteins by autophagy is the SUMOlyation of both proteins.

DNA from pathogens and the cell DNA can be targets of recognition by PRRs [11,44,57]. In eukaryotic cells, self-DNA localization is restricted to the nucleus or mitochondria [156,157]. When self-DNA has been damaged, it moves to the cytoplasm in a structure considered protuberance from the nucleus or mitochondria and is thus recognized as PAMP and/or DAMP by PRRs [16,158,159]. Various stress circumstances can mobilize nDNA or mtDNA to the cytoplasm. For example, in the nucleus during mitosis, poorly segregated chromosomes can be exposed in the cytosol, also due to nuclear rupture due to defects or reduction in the proteins of the structure of nuclear lamina such as Lamin B1, by the generation of protuberance or micronuclei with mutated DNA, and lastly, during DNA leakage due to alterations caused by oxidative stress (Figure 6) [124,142]. From the mitochondria, DNA can also exit the cytoplasm, either due to damage to the mitochondrial membrane, DNA damage, or mutated in conditions of oxidative stress, or when the mitophagy process is altered [11,124,160,161,162].

Currently, more than ten cytosolic receptors for specific intracellular DNA recognition are known, among which we can find the Absent Protein in Melanoma 2 (AIM2), the Regulatory Factors of DNA-Dependent Activating IFNs (DAI), the protein Inducible by Interferon γ 16 (IFI16), RNA Polymerase III and cyclic GMP-AMP Synthase protein (cGAS) [44,163,164]. They all activate signaling pathways that, in some cases, overlap to induce the proinflammatory production of cytokines and molecules in the presence of cytosolic DNA.

cGAS participates in the cGAS/STING signaling pathway, enhancing the immune response after detecting cytoplasmic dsDNA and activating senescence [165].

## 8. The cGAS/STING Signaling Pathway

The cGAS protein is the universal cytoplasmic dsDNA recognition sensor [166,167]. cGAS recognizes the cell’s DNA when damaged and outside its cellular compartments, such as the nucleus and mitochondria [17,165,168] (Figure 6). cGAS also recognizes exogenous DNA such as viral dsDNA, bacterial dsDNA, DNA:RNA hybrids, Y-DNA, and the DNAs considered as DAMP such as mtDNA, nDNA, DNA circulating from tumors cells, extracellular DNA entered into the cell by exosomes or dead cells, damaged and oxidized own DNA [17,165,166,167,168]. The cGAS protein is expressed by a broad spectrum of cells in the body. However, it is mainly present in the immune system cells, such as macrophages, and cells of the physical barriers of our body, such as the skin [17,168].

cGAS binds to DNA in the cytoplasm but is localized during its resting state to the plasma membrane, interacting with the lipid 4,5-phosphatidylinositol bisphosphate [PI(4,5)P2] [169]. In addition, cGAS is present in the chromatin-bound nucleus [168,170]. It acts as a negative regulator of DNA repair, mediated by homologous recombination, which accelerates the destabilization of the genome, the generation of micronuclei, and cell death under conditions of genomic stress [111]. cGAS translocates from the cytoplasm to the nucleus during dsDNA breakage to interact with repair proteins, such as Poly [ADP-ribose] polymerase 1 (PARP1), to prevent homologous recombination [171]. cGAS has an N-terminal tail structure, whose functions are not yet fully understood, and a domain with nucleotidyltransferase (NTase) activity, which catalyzes the synthesis of 2′3′-cGAMP or cGAMP [166,172]. Once cGAS binds to DNA, it forms an oligomeric complex to synthesize cGAMP (Figure 6) [173].

cGAMP is a cyclic dinucleotide whose synthesis depends on GTP and ATP from the cytoplasm [166,174,175]. The synthesis of cGAMP occurs sequentially: (i) cGAS forms 2′-5′ phosphodiester bonds between ATP and GTP to produce the intermediate product pppG (2′-5′)pA, (ii) then induces the formation of phosphodiester bonds between 2′-OH of GMP and 5′-phosphate of AMP and another between 3′-OH of AMP and 5′-phosphate of GMP to produce 2′,3′cGAMP [166,167,172,176]. cGAMP functions as a second messenger, migrating through the cytoplasm and binding to the Stimulator of Interferon Genes (STING) protein [159] (Figure 6). cGAMP can also travel between cells [17]. This occurs across GAP junctions, which directly connect the cytoplasm of neighboring cells and allow the exchange of small molecules between them (Figure 6) [168]. cGAMP can also enter the cytoplasm of another cell via the reduced folate transporter, designated Solute Carrier Family 19 Member 1 (SLC19A1), located on the cell membrane of a neighboring cell (Figure 6) [17,167,177]. This interaction can activate downstream STING signaling and the immune response in neighboring cells [177]. cGAMP can exit the extracellular space and be degraded abroad by phosphodiesterases such as the extracellular protein member of the Ectonucleotide Pyrophosphatase/Phosphodiesterase 1 family (ENPP1) (Figure 6) [17,168,173]. It has been seen that damaged or necrotic cells can also release cGAMP and thus induce paracrine signaling if incorporated into the nearby cell (Figure 6) [168]. cGAMP can be incorporated by cells through structures such as exosomes, which contain the host’s nuclear or mitochondrial DNA [178]. The intercellular transmission of cGAMP implies that this molecule plays a role in the cellular microenvironment so that neighboring cells can activate the immune response by activating STING in the face of a probable viral or bacterial infection (Figure 6) [168].

STING, also known as ERIS, MITA, MPYS, and TMEM173, is a transmembrane protein located in the Endoplasmic Reticulum (RE) that binds cGAMP and other cyclic dinucleotides (CDNs) of bacterial origin, such as dicyclic GMP (CFG) and dicyclic AMP (CDA), so it contributes to both the antiviral and antibacterial response (Figure 6) [179,180,181,182,183,184]. In the resting state, STING is anchored to the Endoplasmic Reticulum (ER) membrane [185]. Once activated, it migrates to the Golgi space, where it associates with TANK-binding protein kinase 1 (TBK1), which phosphorylates STING and allows the recruitment of IRF3 and IkB (Figure 6) [16,17,165,167,174,186]. In its inactive state, STING remains autoinhibited in its C-terminal tail, which is released by ligand binding to interact with TBK1 and IRF3 [57,168,176]. In addition, it has a Ligand Binding Domain that allows the formation of dimers linked by disulfide bridges (at cysteine residues 148) in the form of V; that is, it presents a dimer configuration “open” with a binding site for CDNs located at the bottom of the dimer interface, which closes after binding to cGAMP (Figure 6) [168,184]. For the activation of STING, it must undergo palmitoylation; by the addition of palmitic acid in its cysteine residues close to the transmembrane domains, which facilitates its oligomerization in the Golgi, to produce maximum signal transduction (Figure 6) [17,168,187].

Once phosphorylated, IRF3 forms a homodimer that translocates to the nucleus and activates genes that codes for type I Interferons (I-IFN), including IFN-α and IFN-β (Figure 6) [17]. IFNα and IFNβ are part of the I-IFN family and are effector cytokines of the host’s immune response against viral infections, in addition to presenting immunomodulatory properties [188]. IFNα and β, by binding to and activating the IFN Receptor 1 and 2 (IFNAR 1-2) expressed on the cell surface of a wide range of cell types, generate autocrine and paracrine signaling [189]. This IFNR 1-2 complex activates Janus protein kinase (JAK) and activator of transcription (STAT). When JAK/STAT is activated, IFN-stimulated genes (ISGs) transcription is induced downstream. Once synthesized, the ISGs prevent viral replication, assembly, and release from the interior of infected cells, enhancing the innate and adaptive antiviral immune response [190,191,192]. However, the stimulation of viral immune response is not the only function of I-IFN; these molecules can antagonize the IFN-γ, the only member of Type II IFN, reducing the macrophages response also can block B cells response in bacterial infections inducing immunosuppressive molecules such as IL-10 [193]. Moreover, higher levels of I-IFN can exert an anti-proliferative effect on immune cells with a suppressive and deleterious impact on the immune response [194]. Therefore, their effects can be beneficial or harmful depending on their concentration, stimulation time, and the target cell they stimulate.

Secondly, TBK1 also phosphorylates the IκB kinase so that IκB, which keeps NFκB sequestered, is degraded by the proteasome (Figure 6). Thus, NFκB is free to migrate to the nucleus and induce the transcription of a wide variety of genes associated with the inflammatory process, such as proinflammatory cytokines such as TNF-α, I-IFN, IL-1β, and IL-6, among others (Figure 6) [16,17,167,168,174,186,195].

IRF3 and NFκB are necessary to generate I-INF expression [14]. When cGAS recognizes exogenous DNA and activates STING downstream, considered canonical-type activation, it results in an IRF3-mediated preferential antiviral immune response [14]. Moreover, when nuclear DNA damage occurs, specifically dsDNA breakage, STING forms a complex with p53 and IFI16 in the nucleus. This activation represents a cGAS-independent pathway considered a non-canonical pathway of STING activation [14]. This non-canonical activation induces activation of NFκB principally, leading to an inflammatory response with the expression of a wide variety of cytokines and chemokines [14].

Consequently, after intracellular DNA recognition, the cGAS/STING signaling pathway induces a pro-inflammatory and protective antitumoral response [168].

### 8.1. Regulatory Mechanisms of the cGAS/STING Signaling Pathway

Usually, the cGAS-cGAMP-STING signaling pathway is regulated by four mechanisms: (1) ligand or DNA availability, (2) protein-protein interactions, (3) transcriptional modifications, and 4) degradation of cGAS and STING proteins [165].

To regulate DNA availability, DNA compartmentalization in the nucleus or mitochondria is essential to prevent cGAS activation against self-DNA and avoid developing autoimmunity [156,157]. Aberrant activation of the cGAS/STING pathway coupled with DNase activity deficiency has been linked to various autoimmune diseases, such as Systemic Lupus Erythematosus, Rheumatoid Arthritis, Inflammatory Vascular Disease, and various interferon-associated diseases [196]. The availability of DNA is also regulated by the presence of DNases, which are located in different subcellular compartments (Figure 6). For example, DNase I is located extracellularly, DNase II within the phagolysosome, and DNase III (also known as TREX1) are located in the cytoplasm (Figure 6) [142,165]. When a deficiency in these DNases is generated, damaged DNA is recognized by the cGAS/STING pathway to avoid their accumulation in the cytoplasm and protect the cell from infections or the development of tumors [142,165,197,198].

Activation/deactivation of the cGAS/STING pathway signal is generated by protein-protein interactions and post-transcriptional modifications, which regulate the activity, stability, and/or localization of cGAS and STING proteins (Figure 6) [165]. Among the negative regulation are the acetylation, phosphorylation of cGAS, and the interaction with proapoptotic Caspases 3, 7, and 9, which generates its cleavage and inactivation [199,200,201]. Contrarily, the palmitoylation of STING and its interaction with IFI16 is positive regulation mechanisms of the pathway [165]. As mentioned, cGAMP is also negatively regulated by the phosphodiesterase ENPP1, which generates the breaking of phosphodiester bonds and decreases cGAMP levels and the activation of this pathway (Figure 6) [165].

The elimination of cGAS and STING proteins is generated by degradation through autophagy (Figure 6) [17]. Both proteins are ubiquitinated and packaged into autophagosomes to be digested in autophagolysosomes [165]. Autophagy functions as a negative feedback loop that ensures transient cGAS-STING signaling and prevents over-activation of the pathway [165].

### 8.2. The cGAS/STING Signaling Pathway: Relationship with Senescence and Inflammation Process

The cGAS/STING signaling pathway is highlighted because it is fundamental in regulating cellular senescence induced against DNA damage conditions [18,19,20,32,124,142,168]. In senescent cells occur, a decrease in Lamin B1 levels, the structural protein of the nuclear membrane (Figure 6), induces the release of cytoplasmic chromatin fragments (CCF) into the cytoplasm and, consequently, the activation of the cGAS/STING signaling cascade [18,19,20,202,203,204]. Cells enter a state of senescence due to cell cycle arrest by p53/p21 signaling in response to increased NFκB expression following activation of the cGAS/STING pathway [18,19,20,205]. In this way, these senescent cells’ ROS increase is induced by the response to DNA damage and the activation of the cGAS/STING signaling pathway to stimulate the production of proinflammatory cytokines SASPs through NFκB [18,19,20,206]. The increase in SASPs is necessary for developing the senescent phenotype [18,19,20,124,207]. Therefore, the expression of the cGAS/STING signaling pathway in macrophages and other types of immune cells, together with their participation in the regulation and activation of the senescence, the SAPS secretion, and the activation of the proinflammatory immune response, suggests that this signaling pathway could be involved in the development of immunosenescence and/or inflammation during our lifetime.

### 8.3. The cGAS/STING Signaling Pathway Activation by Oxidized DNA Recognition in Senescent Cells

**Figure 7:**
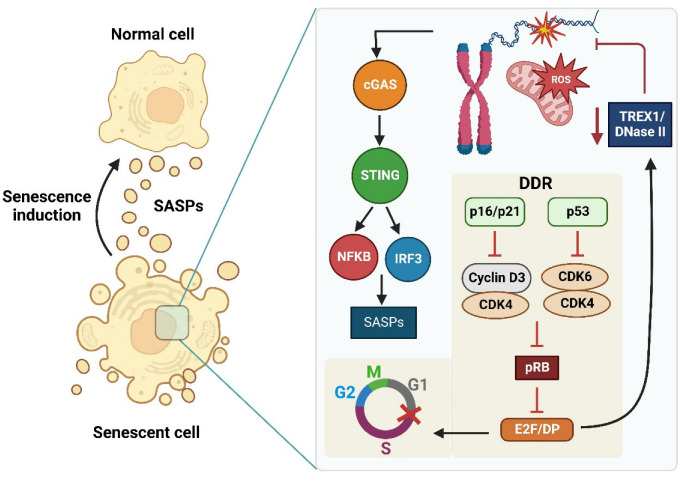
Relationship of the signaling of SASPs, cGAS/STING, ROS, and DDR during senescence. Damaged nuclear or mitochondrial DNA is sensed by cGAS. It activates downstream STING signaling, which generates the increased expression and secretion of SASPs (several types of cytokines such as IL-6, IL-8, and IL-1, among others). SASPs secreted by senescent cells induce senescence activation in the neighbor cells by association with the receptors in the cellular membrane. In addition, damaged DNA causes DDR that activates the cell cycle inhibitors p16/p21 and p53 and the activation of cellular senescence for inhibition of Cyclin-dependent kinases (CDKs) that regulate the cellular cycle. Cytoplasmic DNA can be degraded by DNase II and Three Primer Repair Exonuclease 1 (TREX1), which limits DNA exposure and, thus, cGAS recognition. In addition, the inhibition of the heterodimer of E2F and Dimerization Partner (DP) transcriptional factors, given by Rb, influences the decrease in the expression of nucleases which promotes the accumulation of DNA and the activation of the cGAS/STING pathway during senescence.

The oxidized DNA by ROS is more resistant to degradation by nucleases, which also facilitates its accumulation and the recognition by cGAS, and the activation of the STING pathway together with DDR, which stimulates cell cycle arrest and senescence activation (Figure 7) [16,124,136,142,203,208]. Therefore, the cGAS/STING pathway is essential for cell fate when this pathway recognizes the oxidized DNA since it participates in senescence and regulates cell death [17,205].

The increase in I-IFN levels is produced by the activation of the cGAS/STING signaling pathway in senescent cells exposed to oxidative stress [202,203,204]. It suggests that the ROS can induce the activation of the cGAS/STING signaling pathway. In addition, molecules such as 8-hydroxyguanosine (8-OH-dG), an oxidative derivative of guanosine that induces DNA adducts and oxidized DNA, a potent activator of the cGAS/STING pathway [203]. All this information suggests that ROS’s modifications to DNA would be recognized by the cGAS/STING signaling pathway, activating its signaling and enhancing the senescence induction and the inflammatory response. The senescence increases, and DNA damage by ROS is a classic cellular effect that triggers aging and augments progressively during life, inducing Inflammaging (Figure 7). Hence, the cGAS/STING signaling pathway presumably participates in the Inflammaging regulation. However, this has not been proven. Further studies are needed to demonstrate this and the relationship between damaged DNA, ROS, cGAS/STING signaling, and Inflammaging during life.

## 9. Conclusions and Future Perspectives

**Figure 8:**
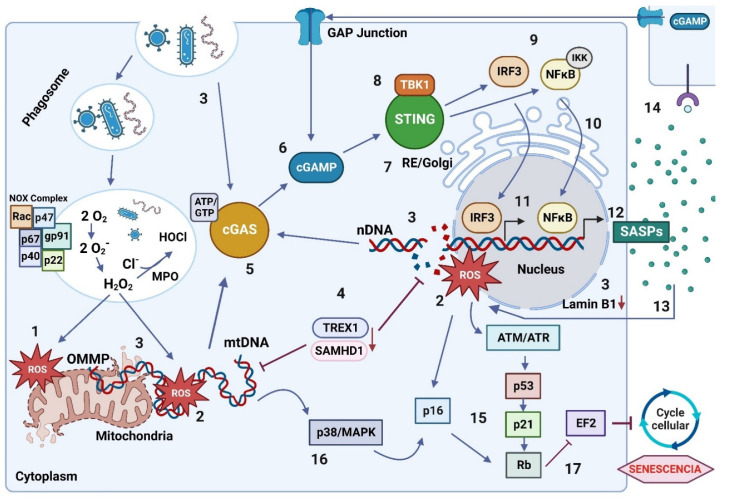
Proposed Relationship Between the cGAS/STING Signaling Pathway and ROS During Inflammaging in the Senescent Macrophage. (1) Increased ROS production is generated from sources such as mitochondria and oxidative burst (2) ROS induce damage to mitochondrial and nuclear DNA (3) Damaged mtDNA is located in the cell cytoplasm, moving from the interior of mitochondria due to increased Outer Mitochondrial Membrane Permeabilization (OMMP) and nuclear DNA moves from the nucleus to the cytoplasm due to decreased structural protein Lamin B1. In addition, the accumulation of DNA in the cytoplasm is generated by alterations in the function of the degrading enzymes DNase II and TREX1 (4). The sensor protein cGAS recognizes nuclear and mitochondrial DNA in the cytoplasm (5). After binding with DNA, cGAS is activated and produces 2′3′cGAMP from GTP and ATP. cGAMP has the ability to exit the cell and enter another neighboring cell through GAP junctions (6) cGAMP binds to STING in the ER and STING moves to the Golgi (7) STING forms a complex with TBK1 which phosphorylates it (8) TBK1 generates the parallel phosphorylation of IRF3 and IKK (9) IKK phosphorylates IκB and NFκB remains free (10) IRF3 and NFκB translocate to the nucleus (11) IRF3 and NFκB initiate transcription of genes such as IFN I and IL-6, IL-8 and TNF α, respectively (not shown in the figure), which together form part of the SASPs (12) The SASPs induce an increase in ROS, which increase oxidative damage, generating a positive feedback loop on the activation of the cGAS/STING pathway (13) SASPs released in the cell are able to reach neighboring cells to induce senescence, inflammation and ROS in the tissue (14) DNA damage induces DDR (as mentioned in the text), with activation of p53-21 and p16 to induce a Rb (15) ROS induce MKK3/MKK6 signaling pathway (not shown) for activation of p38 MAPK (16) p38 MAPK activates p53 and p16 for the induction of Rb (17) Rb generates the inhibition of the EF2 factor, which produces irreversible cell cycle arrest and activation of senescence.

Predictions from the WHO indicate that around 30% of the world population of planet’s inhabitants will exceed 60 years of age around the year 2050 [1]. Therefore, the study of mechanisms that allow healthy aging and pathologies associated with age must be evaluated from now on. Hence, studying the regulatory systems of the body’s homeostasis during aging, such as the immune system, is essential [3]. The aging of the immune system or Immunosenescence and Inflammaging process are generated principally by the deregulation of immune cells and their functions with the increase in inflammatory and oxidative molecules [5]. Macrophages correspond to cells that are high producers of pro-inflammatory cytokines and ROS, which makes them the main actors in Inflammaging [4]. The increased production of ROS translates into a loss of redox balance, which induces oxidative damage to the essential macromolecules of a cell, such as DNA (Figure 8) [68,69]. DNA damage triggers signaling pathways to activate the cell cycle arrest, triggering the senescence process to protect the organism from potential tumor cells [124].

Under the activation of senescence, the release of SASPs by cells is generated, which promotes an inflammatory microenvironment and induces a more significant generation of ROS, which becomes a vicious circle [124,128,142]. Among the signaling pathways that participate in the positive regulation of the release of SASPs and the activation of senescence is the cGAS/STING signaling pathway, which after recognition of cytosolic DNA, is activated (Figure 8) [18,19,20,124]. The increase in SASPs triggered by the cGAS/STING pathway promotes senescence activation in neighboring cells due to paracrine communication through SASPs [32,132,133]. In addition, direct communication between cells is generated through GAP junctions, which are used by the second messenger cGAMP pathway to transmit the pathway signal through the tissue (Figure 8) [172,177]. This could imply the propagation of the proposed loop between the activation of the cGAS/STING pathway and the increase in ROS (induced by SASPs) and, therefore, the amplification of Inflammaging by the tissue.

The increase in ROS from mitochondria by the oxidative burst is a phenomenon that occurs during life in the immune cells in the presence of pathogens. However, during aging, this process is deregulated as a consequence of the oxidative DNA damage, which triggers the activation of cellular senescence through various mechanisms and pathways: the DDR, the p38 MAPK pathway, and the activation of the cGAS/STING signaling pathway (Figure 8) [16,19,203]. In addition, in aging, the nuclear DNA accumulates in the cell cytoplasm due to the decrease in Lamin B1 expression (Figure 8) [121,124]. In addition, there is an alteration in the function of DNA-degrading nucleases, which facilitates the accumulation of damaged DNA in the cytoplasm for its recognition by the cGAS/STING pathway [18,19,20,124,142]. Moreover, in the face of nuclear DNA damage, cGAS-independent signaling is generated that activates STING, which forms a complex with p53 and IFI16, which preferentially activates the transcription mediated by NFκB over IRF3 and stimulates the release of cytokines and molecules proinflammatory [14], which could indicate an essential involvement of STING in the regulation of Inflammaging.

The cGAS/STING signaling pathway is expressed in various cell types but mainly in antigen-presenting cells, such as macrophages. When activated in these cells, it regulates the innate and adaptive immune response against pathogens [168]. ROS play an indirect but essential role in the activation of the cGAS/STING pathway since they increase the formation of nuclear and mitochondrial DNA oxidation products, which are recognized by this pathway [202,203,204]. However, the relationship between ROS, DNA damage, and the activation of the cGAS/STING signaling pathway during the inflammatory process has not been described. For this reason, in this review, we propose that the cGAS/STING signaling pathway plays a fundamental role in regulating Inflammaging when oxidative damage occurs in DNA due to an increase in ROS (Figure 8). We propose a positive loop between the activation of the cGAS/STING pathway and the increase in ROS with DNA damage during Inflammaging. Consequently, the cGAS/STING pathway will activate senescence and release SASPs that perpetuate the senescence in other cells. (Figure 8), which induces ROS and increases oxidative DNA damage (Figure 8). However, even though both senescence and increased oxidative stress are characteristic of Inflammaging, there is no clear information on the role played by the cGAS/STING pathway during the aging process and its relationship with the increase in ROS. Understanding the molecular mechanisms of the cGAS/STING pathway and ROS and how they are coordinated is essential to clarify an important part of the aging process.

A relationship has been described between the reduction of oxidative stress and the decrease in the activation of the cGAS/STING pathway through ROS regulatory molecules [206,209]. The antioxidant enzyme Glutathione Peroxidase 4 (GPX4) protects membrane lipids from oxidation by the action of ROS since it reduces oxidized fatty acids and lipid hydroperoxides, therefore, increasing lipid peroxidation. Consequently, forming these lipid peroxidation products leads to the carbonylation of STING at C88, which inhibits the palmitoylation of this protein that is necessary for the translocation from ER to the Golgi and, as a consequence, prevents it signaling [206]. In the same way, it has been shown that the Erythroid-derived Nuclear Factor 2 (Nfr2), involved in the detoxification of ROS through the transcription of antioxidant enzyme genes, suppresses the expression of STING and negatively regulates the inflammatory and antiviral response [209]. Recently, a new regulatory mechanism between ROS and the activation of the cGAS/STING signaling pathway has been proposed that involves a reduction in the activation of this pathway by ROS. Tao et al. [210]. demonstrated that the oxidation of cysteine 147 of STING by ROS prevents the polymerization of this protein and, consequently, the inhibition of its signaling and the secretion of type I-IFNs [210]. These data indicate that the relationship between the oxidation products by ROS and the cGAS/STING signaling pathway is not clear, and to clarify it, the cell type, the ROS levels, and, in addition, the time or the moment of the life where the process is occurring.

In recent years, the cGAS/STING pathway has been studied for therapeutic purposes [211]. Knowledge about this pathway has been directed to treat mainly inflammatory diseases, such as autoimmune and potential immunological therapies against cancer [165]. Rapid progress has been made in finding STING agonists, such as 5,6-dimethylxanthenone-4-acetic acid (DMXAA) and 10-carboxymethyl-9-acrydanone (CMA), capable of generating immunostimulatory, antitumor, and antiviral effects [211,212,213]. However, they have low permeability to enter cells and have also been found to be specific for murine, so they have not been successful in humans [211,212]. Several analogs have recently been tested as new STING receptor agonists for humans and other species, with good results and functions similar to cGAMP [211,214] Some examples of STING antagonists are tetrahydroisoquinolines, which are small molecules that bind the CD-binding domain of STING with low affinities, such as the Compound **18** (C-**18**) [215]. Astin C is a natural product that binds competitively with the CDN site of STING, similar to C-**18**. The nitrofurans C-**170** and C-**171** with a modification of butyl and hexyl alkyl groups at the 4-position of the phenyl ring inhibited both human and mouse STING [213,215]. Nitro fatty acids such as nitrolinoleic acid and nitrooleic acid covalently modify STING in Cys88 and Cys91 in human fibroblasts [213,215]. Acrylamides BPK-21 and BPK-25 formed adducts with Cys91 of STING, preventing palmitoylation and downstream signaling [213,215]. SN-011, by association with CDN, impedes STING oligomerization, trafficking, and signaling activation in the presence of cytosolic DNA. SN-011 inhibits the secretion of pro-inflammatory molecules in vitro mouse and human models, including HSV-1 infection and *Trex1* deficiency in mice [216]. Like SN-011, the H-151 inhibits STING with similar IC_50_ in mouse cell lines. H-151 is associated with Cys91 of STING in the transmembrane domain [213,216]. All these molecules could reduce the cGAS/STING signaling in different cells and tissue. However, most STING inhibitors mentioned and others have been tested only in mouse and human-origin cell lines. In some cases, their effectiveness has been proven in animals. None have been tested on aging models or to inhibit the Inflammaging process or senescent markers, and in no case have their side effects been analyzed. In addition, most of the research on the cGAS/STING pathway has been focused on I-IFN effects in different processes and their participation in the antiviral response without considering the contribution of the cGAS/STING pathway canonical and non-canonical in the regulation of senescence and the activation of proinflammation and ROS secretion [16,17,217]. Moreover, although it is known that the cGAS/STING pathway regulates senescence induced by oxidative stress, no studies prove this relationship during aging [19]. It is even unknown if there are changes in the levels of the proteins that make up the cGAS/STING pathway during aging in different tissues. All information exposes in this review indicates that activating the cGAS/STING pathway generates the molecular link between nuclear or mitochondrial DNA damage, ROS increase, senescence, and probably the Inflammaging generation during life. Therefore, the cGAS/STING signaling pathway has a leading role in the development of inflammation and its participation in the cellular and molecular changes that occur during aging, which should be analyzed and taken into account in the future. We think that the study of the role of the cGAS/STING signaling pathway is crucial during life and in health and diseases because.

## Figures and Tables

**Table 1:** Biological sources of ROS generation.

Exogenous Sources	Refs.	Endogenous Sources (Enzymatic)	Refs.
Ionizing radiation Non-ionizing radiation Ozone (O_3_) Tobacco smoke Environmental pollutants Pesticides Heavy metals	[71,72] [73] [73] [74] [75] [76] [74]	Mitochondrial Respiratory Chain p450 cytochrome (Endoplasmic Reticulum) Xanthine Oxidase Nitric Oxide Synthase NADPH Oxidase	[77] [78] [73] [73] [79]
